# Causal effect of diabetes duration on productivity by socio-economic position in Germany between 2009 and 2021

**DOI:** 10.1093/eurpub/ckag041

**Published:** 2026-03-24

**Authors:** Malwina M Mackowiak, Annika Hoyer, Katharina Piedboeuf-Potyka, Markus Neuhäuser, Oliver Kuss, Thaddäus Tönnies

**Affiliations:** RheinAhrCampus, Department of Mathematics, Informatics, Technology, Koblenz University of Applied Sciences, Remagen, Germany; Medical School OWL, Biostatistics and Medical Biometry, Bielefeld University, Bielefeld, Germany; Institute for Biometrics and Epidemiology, German Diabetes Center (DDZ), Leibniz Center for Diabetes Research at Heinrich Heine University, Düsseldorf, Germany; RheinAhrCampus, Department of Mathematics, Informatics, Technology, Koblenz University of Applied Sciences, Remagen, Germany; Institute for Biometrics and Epidemiology, German Diabetes Center (DDZ), Leibniz Center for Diabetes Research at Heinrich Heine University, Düsseldorf, Germany; German Center for Diabetes Research, Partner Düsseldorf, München-Neuherberg, Germany; Centre for Health and Society, Medical Faculty and University Hospital, Heinrich-Heine-University, Düsseldorf, Germany; Institute for Biometrics and Epidemiology, German Diabetes Center (DDZ), Leibniz Center for Diabetes Research at Heinrich Heine University, Düsseldorf, Germany

## Abstract

Diabetes negatively impacts productivity, but the extent to which socio-economic factors influence this effect is unknown. This study examines how diabetes duration affects labour force participation and sick leave in Germany, focusing on socio-economic differences. We used self-reported data collected between 2009 and 2021 from the German Socio-Economic Panel Study, a longitudinal household survey. People with prevalent diabetes at baseline were excluded. To estimate the causal effect of diabetes duration on the outcomes, we employed marginal structural regression models for repeated measures, using stabilized inverse-probability-of-treatment-and-censoring weights to adjust for informative censoring, time-fixed (sex, age, socio-economic position, migration background) and time-varying confounding (body mass index, physical activity frequency, smoking status, previous outcome). We included interaction terms to assess diabetes-related productivity losses by subgroups of socio-economic position, sex, age and migration background. The analysis consisted of 35 906 observations from 18 456 individuals for the outcome labour force participation and 12 469 observations from 7244 individuals for the outcome sick leave days. A five-year increase in diabetes duration was associated with a labour force participation shortfall of 13.8% (95% confidence interval: 5.8; 21.1) and an increase of 6.8 sick leave days (-5.4; 19.0). Effects were more pronounced among individuals in lower socio-economic position and diminished with increasing socio-economic position. Diabetes-associated productivity losses predominantly affect people in low socio-economic position, reflecting a dual burden of higher diabetes prevalence and larger productivity losses.

## Introduction

Diabetes mellitus imposes a substantial economic burden at both individual and population levels by negatively affecting productivity. Globally, the economic burden of diabetes was estimated at 1.8% of the gross domestic product in 2015, with 40% of these costs attributed to morbidity-associated production and productivity losses in high-income countries [1]. In particular, reduced labour force participation (LFP), premature withdrawal from labour market and early retirement are major contributors to these losses [2]. In Germany, where pension entitlements primarily depend on the length and amount of income over a person’s working life [3], individuals with diabetes may face an elevated risk of income poverty in old age given the reduced productivity during their working years. At the population level, the projected rise in diabetes prevalence, coupled with its detrimental effects on labour market outcomes, suggests an increasing economic burden in Germany [4–6].

Diabetes prevalence is not evenly distributed across socio-economic strata. Individuals in lower socio-economic position (SEP), defined by education, occupation and income, are more often affected by the disease, globally [7] and within Germany [8, 9]. A systematic review reported overall relative risks (RRs) for developing type 2 diabetes of 1.41 (95% confidence interval, CI: 1.28; 1.51), 1.31 (95% CI: 1.09; 1.57) and 1.40 (95% CI: 1.04; 1.88) when comparing people with a low and high education, occupation and income, respectively [7]. Despite this evidence, few studies have examined whether productivity losses associated with diabetes are disproportionately higher among individuals in lower SEP. Understanding this association is essential, as it may reveal a dual disadvantage – greater diabetes risk and more severe economic consequences – for people in lower socio-economic groups. Therefore, the primary objective of this study was to estimate the causal effect of diabetes duration on the two crucial productivity measures LFP and sick leave days (SLD), overall and across socio-economic subgroups.

## Methods

### Data source and study design

To investigate the effect of diabetes duration on LFP and SLD, we used longitudinal data between 2009 and 2021 from the German Socio-Economic Panel Study (GSOEP), a nationally representative household survey primarily based on face-to-face interviews [10]. We excluded individuals with self-reported diabetes at baseline and included participants with at least one follow-up interview, living in private households and aged 20–69 years, assuming this to be the relevant working age range. Diabetes status was first recorded in 2009, with the latest available data from 2021 at the time of our analysis. As diabetes-related questions were collected biennially, our study comprised data from one to six subsequent biennial follow-up interviews for each individual. Further GSOEP details are provided by Goebel et al. [10] and the web-based documentation [11]. This study was approved by the Ethics Committee of Heinrich Heine University Düsseldorf, Germany (reference 2022-2219).

### Variables

In this study, we adhered to the International Labour Organization’s [12] definition of LFP. Participants were considered part of the labour force if they had engaged in paid employment during the seven days before the interview or were unemployed, but available for work and actively seeking employment within the previous four weeks. Accordingly, individuals outside the labour force had not engaged in paid work in the preceding seven days and either lacked future employment intentions or could not start acceptable work within two weeks without recent job search efforts in the prior four weeks. The self-reported number of days a person was unable to work due to illness in the corresponding calendar year was operationalized as SLD. The presence or absence of diabetes was determined based on participants’ responses to the question “Has a doctor ever diagnosed you to have one or more of the following illnesses?,” without differentiation by diabetes type [13]. Once this question was answered with “yes,” we assumed that the diabetes status did not change thereafter. As the GSOEP relies on self-reported information, the inclusion of laboratory-based measures, such as HbA1c, or clinically verified diabetes diagnoses was not possible for assessing diabetes status. SEP was determined using a revised version of the socio-economic status index described in Lampert et al. [14]. This index combines information on education, occupation and income into a score ranging from 3.0–21.0, with lower values indicating a lower SEP. The educational level reflects the highest individual educational qualification, the occupational level the highest household occupational position, and income the gross equivalised disposable household income [14]. In the GSOEP, migration background was assessed based on the country of birth of the individual or their parents. Individuals born outside Germany were classified as having a direct migration background. Those born in Germany with at least one parent born abroad were classified as having an indirect migration background. All others were classified as having no migration background. We analysed diabetes status (present, absent), LFP (yes, no), sex (female, male), smoking (yes, no), physical activity frequency (almost never or never, several times a year, at least once a month, at least once a week), migration background (direct or indirect, none), and baseline year (2009, 2011, 2013, 2015, 2017, 2019) as categorical variables. SLD, SEP, diabetes duration, age, BMI, and follow-up interview indicator were modelled as continuous variables.

### Statistical analysis

**Figure 1.**
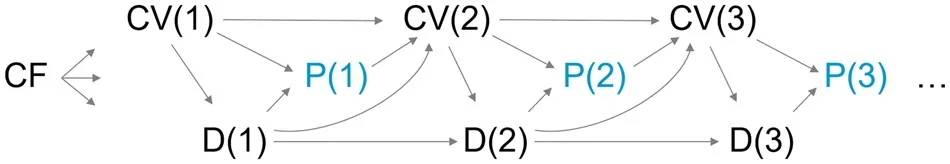
Directed Acyclic Graph illustrating dependencies between time-fixed confounders *CF*, time-varying confounders *CV*(*t*), diabetes status *D*(*t*) and outcome *P*(*t*) across follow-ups *t* (*t* = 1, …, 6). Time-fixed confounders *CF* are baseline variables that affect all other variables at all follow-ups.

Since time-varying confounders may affect productivity measures, predict subsequent diabetes and simultaneously be influenced by previous diabetes, standard statistical methods may yield biased estimates for the causal effect. To address this issue, we employed marginal structural regression models for repeated measures, which allow for the appropriate adjustment of such confounders [15]. As a result, consistent estimates of the causal effect of diabetes duration on LFP and SLD were obtained. To adjust for confounding and informative censoring, we applied stabilized inverse-probability-of-treatment-and-censoring (IPTC) weights as described by Hernán et al. [16]. We selected variables to calculate stabilized IPTC weights based on existing literature and data availability in the GSOEP. Thereby, we assumed that the selected variables suffice to adjust for confounding and mitigate selection bias arising from loss to follow-up. The outcomes of interest in the marginal structural models (MSMs) were LFP (*P_1_*(*t*)) and SLD (*P_2_*(*t*)). BMI, physical activity, smoking and previous outcome were considered as time-varying confounders *CV*(*t*), (*t* = 1,…,6). Sex, age, SEP and migration background were considered as time-fixed confounders *CF* obtained at baseline (*t = 0*), i.e. before the start of follow-up. We defined baseline as earliest observation of an individual having information on diabetes, sex, age, migration background, BMI, smoking, physical activity and LFP or SLD, respectively. A person was right-censored, if at least one variable had missing information, if the person died or reached the administrative end of follow-up. We did not account for death as a competing risk since only 0.5% of participants died during follow-up. Given potential differences in annual GSOEP interview questions, we ensured that information on confounders *CV*(*t*) preceded information on diabetes status *D*(*t*) by using values from the previous year or two years prior, whichever was available and closest to the relevant follow-up. Throughout, we assumed that only the most recent confounder values *CV*(*t*) affect *D*(*t*) (Fig. 1). Further details on IPTC weight calculation appear in the supplementary material.

In our MSMs, we estimated the RRs of LFP-shortfall and SLD-increase with diabetes duration, baseline variables *CF*, follow-up interview indicator *t* and baseline year as independent variables. LFP-shortfall was estimated using a marginal structural Poisson model allowing for over- and underdispersion. The increase in SLD was estimated using marginal structural linear regression. Both MSMs employed robust variance estimation to account for weighting and within-subject correlation. Relative LFP-shortfall [%] was calculated as (1—RR) × 100. In further models, we included an interaction term between diabetes duration and SEP, sex, age or migration background to allow for subgroup-specific associations between diabetes duration and the outcomes. Following Lampert et al. [14], SEP-specific results are reported for the midpoints of the SEP groups low (below the first quintile, midpoint = 7.0), middle (first to fourth quintile, midpoint = 11.0), and high (above the fourth quintile, midpoint = 16.0). These subgroups were based on stabilized IPTC-weighted quintiles of the empirical SEP distribution.

To assess the robustness of our findings, we conducted multiple sensitivity analyses covering IPTC-weight distribution and stability, variable balance, unmeasured confounding, the COVID-19 pandemic and the impact of missing data. Further details are provided in the supplementary material.

The description of this study adhered to the STROBE guidelines for observational studies [17]. All statistical analyses were conducted using SAS version 9.4. (SAS Institute Inc., Cary, NC, USA), with MSMs estimated via PROC GENMOD. Sensitivity analysis procedures and macros are detailed in the supplementary material. Figures were created using R version 4.4.1 (The R Foundation for Statistical Computing, Vienna, Austria) and Microsoft PowerPoint.

## Results

The LFP study population comprised 35 906 observations from 18 456 participants, excluding 1712 observations with missing data in the analysis variables. The SLD study population comprised 12 469 observations from 7244 participants after excluding 9838 observations with missing data. Descriptive statistics of the study populations are reported in Tables 1 and 2, respectively.

**Table 1.** Characteristics of study population for outcome labour force participation over time

	Follow-up (*t*)							
	0 (*N* = 18 456)	1 (*N* = 18 456)	2 (*N* = 8246)	3 (*N* = 4902)	4 (*N* = 2271)	5 (*N* = 1391)	6 (*N* = 640)	Total (*N* = 54 362)
Diabetes, *n* (%)	0 (0.0%)	260 (1.4%)	245 (3.0%)	203 (4.1%)	136 (6.0%)	112 (8.1%)	58 (9.1%)	1014 (1.9%)
Labour force participation, *n* (%)	15 318 (83.0%)	15 316 (83.0%)	7031 (85.3%)	4167 (85.0%)	1902 (83.8%)	1129 (81.2%)	481 (75.2%)	45 344 (83.4%)
Women, *n* (%)	9903 (53.7%)	9843 (53.3%)	4192 (50.8%)	2429 (49.6%)	1038 (45.7%)	643 (46.2%)	287 (44.8%)	28 335 (52.1%)
Age [years]								
*N*	18 456	18 456	8246	4902	2271	1391	640	54 362
Mean (SD)	43.8 (12.7)	45.8 (12.6)	47.8 (11.5)	50.3 (10.8)	52.6 (10.6)	54.3 (9.9)	56.6 (9.1)	46.4 (12.5)
Socioeconomic status index								
*N*	18 456	18 446	8241	4900	2270	1391	640	54 344
Mean (SD)	12.1 (3.9)	12.5 (3.8)	12.8 (3.7)	13.2 (3.6)	13.2 (3.4)	13.5 (3.4)	13.5 (3.5)	12.6 (3.8)
Body mass index								
*N*	18 456	18 456	8246	4902	2271	1391	640	54 362
Mean (SD)	25.8 (4.7)	26.1 (4.8)	26.5 (4.9)	26.7 (5.0)	27.0 (5.1)	27.2 (5.1)	27.4 (5.4)	26.2 (4.8)
Smoking, *n* (%)	5734 (31.1%)	5520 (29.9%)	3226 (39.1%)	1961 (40.0%)	1064 (46.9%)	617 (44.4%)	264 (41.3%)	18 386 (33.8%)
Frequency of exercise, *n* (%)								
Almost never or never	5577 (30.2%)	6090 (33.0%)	2841 (34.5%)	1374 (28.0%)	754 (33.2%)	419 (30.1%)	138 (21.6%)	17 193 (31.6%)
Several times a year	3241 (17.6%)	3122 (16.9%)	1401 (17.0%)	839 (17.1%)	387 (17.0%)	276 (19.8%)	131 (20.5%)	9397 (17.3%)
At least once a month	1243 (6.7%)	1249 (6.8%)	526 (6.4%)	283 (5.8%)	153 (6.7%)	79 (5.7%)	30 (4.7%)	3563 (6.6%)
At least once a week	8395 (45.5%)	7995 (43.3%)	3478 (42.2%)	2406 (49.1%)	977 (43.0%)	617 (44.4%)	341 (53.3%)	24 209 (44.5%)

**Table 2.** Characteristics of study population for outcome sick leave days over time

	Follow-up (*t*)						
	0 (*N* = 7244)	1 (*N* = 7244)	2 (*N* = 3588)	3 (*N* = 966)	4 (*N* = 434)	5 (*N* = 237)	Total (*N* = 19 713)
Diabetes, *n* (%)	0 (0.0%)	78 (1.1%)	72 (2.0%)	29 (3.0%)	20 (4.6%)	14 (5.9%)	213 (1.1%)
Sick leave [days/year]							
*N*	7244	7244	3588	966	434	237	19 713
Mean (SD)	8.2 (22.4)	11.4 (31.4)	12.4 (33.4)	14.2 (35.4)	13.8 (36.8)	16.9 (44.0)	10.7 (29.5)
Women, *n* (%)	3637 (50.2%)	3789 (52.3%)	1824 (50.8%)	456 (47.2%)	189 (43.5%)	101 (42.6%)	9996 (50.7%)
Age [years]							
*N*	7244	7244	3588	966	434	237	19 713
Mean (SD)	42.3 (11.4)	43.8 (11.0)	46.5 (10.1)	49.2 (10.4)	50.7 (9.8)	53.1 (8.7)	44.3 (11.1)
Socioeconomic status index							
*N*	7244	7240	3588	966	434	237	19 709
Mean (SD)	12.5 (3.7)	12.9 (3.7)	13.5 (3.5)	13.2 (3.4)	13.4 (3.3)	13.8 (3.3)	12.9 (3.7)
Body mass index							
*N*	7244	7244	3588	966	434	237	19 713
Mean (SD)	25.5 (4.4)	26.0 (4.8)	26.4 (4.8)	26.8 (5.0)	26.6 (4.6)	27.0 (4.7)	26.0 (4.7)
Smoking, *n* (%)	2332 (32.2%)	2275 (31.4%)	1198 (33.4%)	485 (50.2%)	215 (49.5%)	108 (45.6%)	6613 (33.5%)
Frequency of exercise, *n* (%)							
Almost never or never	1657 (22.9%)	2486 (34.3%)	1184 (33.0%)	336 (34.8%)	160 (36.9%)	74 (31.2%)	5897 (29.9%)
Several times a year	1526 (21.1%)	1069 (14.8%)	525 (14.6%)	150 (15.5%)	61 (14.1%)	41 (17.3%)	3372 (17.1%)
At least once a month	651 (9.0%)	445 (6.1%)	197 (5.5%)	51 (5.3%)	22 (5.1%)	9 (3.8%)	1375 (7.0%)
At least once a week	3410 (47.1%)	3244 (44.8%)	1682 (46.9%)	429 (44.4%)	191 (44.0%)	113 (47.7%)	9069 (46.0%)

Among the 18 456 individuals of the LFP study population interviewed at baseline and follow-up 1, participation in follow-up interviews 2–6 was 45%, 27%, 12%, 8%, and 3%. Observations were primarily excluded due to missing data for BMI, physical activity, smoking, diabetes or LFP. Including only participants without diabetes at baseline, the percentage of people with diabetes increased by an average of 1.5 percentage points (pp) between consecutive follow-up interviews. LFP varied between 75.2% and 85.3% with a total average of 83.4%. The proportion of women decreased from 53.7% at baseline to a minimum of 44.8% at follow-up 6.

In the SLD study population, participation was 49% at follow-up 2, 13% at follow-up 3, 6% at follow-up 4 and 3% at follow-up 5. Follow-up 6 data were unavailable as SLD were recorded retrospectively. Exclusions were mainly due to incomplete data on BMI, physical activity, smoking, diabetes or SLD. The percentage of people with diabetes increased at a slower rate than in the LPF study population with an average of 1.2 pp between consecutive follow-ups. The mean number of SLD increased from a minimum of 8.2 days per year at baseline to a maximum of 16.9 at follow-up 5. The decline in the proportion of women was less pronounced than in the LFP study population.

SEP and BMI exhibited an increasing trend over time in both study populations. Smoking prevalence also rose, by up to 18.0 pp relative to follow-up 1, which had the lowest levels. Physical activity showed no clear temporal trend, with most participants reporting activity at least once per week (44.5% or 46.0%) or almost never/never (31.6% or 29.9%). The distribution and descriptive statistics of diabetes duration are presented in Fig. S1 of the supplementary material.

**Figure 2.**
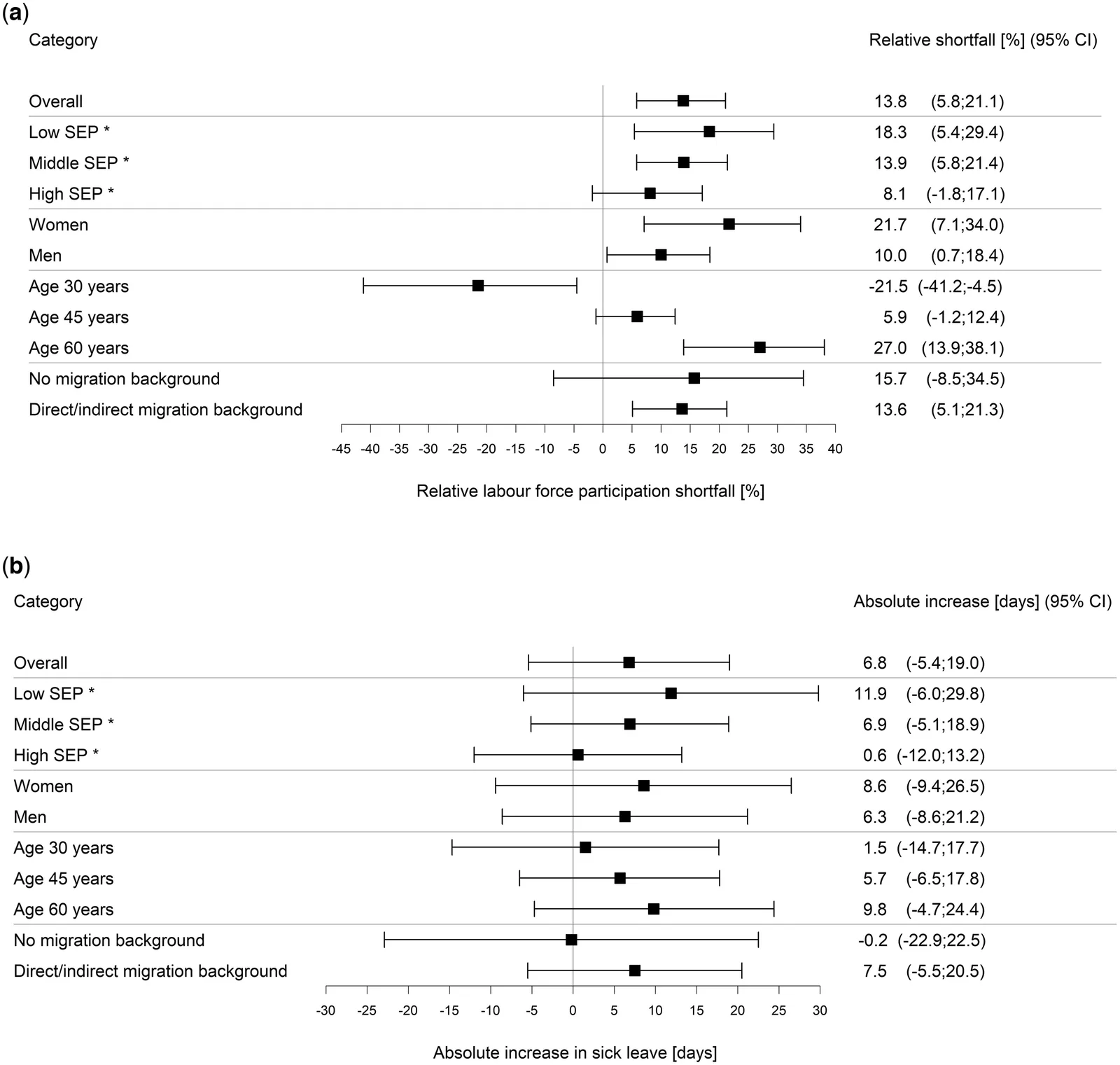
Relative labour force participation shortfall (a) and absolute increase in sick leave days (b) associated with five additional years of diabetes duration overall and by subgroups. Results are based on marginal structural regression models for repeated measures adjusted for time-fixed and time-varying confounding using Poisson regression (a) and linear regression (b). * SEP, socio-economic position.

The causal effect estimate, derived from the MSM modelling the effect of diabetes duration on LFP, indicated a 13.8% reduction in LFP (95% CI: 5.8; 21.1) associated with five additional years of diabetes duration (Fig. 2a). The average shortfall was highest among individuals in lower SEP and progressively declined with higher SEP, leading to estimates of 18.3% (95% CI: 5.4; 29.4), 13.9% (95% CI: 5.8; 21.4), and 8.1% (95% CI: -1.8; 17.1) in the categories low, middle and high. Generally, LFP-shortfall was more pronounced in women and older individuals. Regarding migration background, the effect was similar across the two strata.

The causal effect estimate, obtained through a MSM assessing the relationship between diabetes duration and SLD, indicated that a five-year increase in diabetes duration is associated with an increase of 6.8 SLD (95% CI: -5.4; 19.0). As illustrated in Fig. 2b, the MSM-derived estimates demonstrated heterogeneity across SEP categories. The increase in SLD was highest among individuals in lower SEP and progressively declined with higher SEP, leading to estimates of 11.9 (95% CI: -6.0; 29.8), 6.9 (95% CI: -5.1; 18.9), and 0.6 (95% CI: -12.0; 13.2) in the SEP groups low, middle and high, respectively. Generally, the increase in SLD was more pronounced among women and older individuals, consistent with findings from the LFP study population. However, all estimates based on the SLD study population exhibited lower precision and extensively overlapping CIs, limiting the strength of the evidence. Due to high variability in the group with no migration background, no conclusions can be drawn regarding differences across migration background strata.

Generally, sensitivity analyses supported our key results, confirming a negative impact of diabetes on productivity, with increasing losses observed with decreasing SEP. However, some differences in sex and age-specific associations were attenuated or disappeared in sensitivity analyses with multiple imputation. For instance, the sex-related gradient observed in the main analysis was markedly reduced in the LFP study population and absent in the SLD study population. Further details are provided in the supplementary material.

## Discussion

### Summary of findings

Our study revealed that the relative shortfall in LFP and the absolute increase in SLD were generally higher among people in low SEP and decreased with increasing SEP. Negative effects on both productivity measures were more pronounced among women and older individuals in the main analysis.

These findings corroborate the results of our earlier analysis, which examined the probability of labour force participation by diabetes status and the associated LFP-shortfall across strata of sex, age and SEP. That prior analysis was exploratory in nature, as it did not adjust for potential confounders and did not make use of the longitudinal design of the GSOEP data [18].

Our results also align with other literature on diabetes-associated productivity losses. Specifically, we found that five additional years of diabetes duration were associated with an estimated LFP-shortfall of 13.8% (95% CI: 5.8; 21.1) and an SLD-increase of 6.8 (95% CI: -5.4; 19.0). These estimates support prior research reporting a negative association between diabetes and productivity, measured by indirect costs and productivity-adjusted life years lost [5, 19].

Sex-specific analyses revealed greater adverse effects of diabetes on productivity among women than men. Our LFP-shortfall estimates (10.0% for men and 21.7% for women) were slightly lower than those reported by Bommer et al. [1] for high-income countries (12.6% for men and 25.2% for women). Moreover, their absenteeism findings showed a considerably lower number of SLD compared to our study. Both observations suggest the need for country-specific analyses for assessing the productivity burden of diabetes. Although consistent with previous studies, sex differences attenuated or disappeared after multiple imputation for missing data in sensitivity analyses. This indicates that these differences may result from selection bias rather than from true differences, so sex-specific results should be interpreted with caution.

Additionally, our study supports the findings that diabetes-associated productivity losses are higher among older individuals, consistent with Pedron et al. [2], who linked diabetes to an increased likelihood of early retirement. However, these age-specific differences in the estimates should be viewed cautiously as they may reflect selection bias, given that they attenuated when we accounted for missing data using multiple imputation in sensitivity analyses.

Regarding socio-economic inequalities in the economic burden of diabetes, our results are in line with those of He et al. [20], who reported that the fastest rise in type 2 diabetes-related mortality and disability-adjusted life years occurred in low and low-middle socio-demographic index regions.

### Implications for public health

People in low SEP experience a higher risk of diabetes [7] and more severe diabetes-associated productivity losses, leading to disproportionate high LFP-shortfall and SLD-increase within this subgroup. Beyond disease burden, these individuals face exacerbated economic consequences, including reduced pension entitlements and elevated risks of income poverty during older age, compared to their counterparts in high SEP. Diabetes has wide ranging societal effects in Germany, making it a major public health concern of economic relevance. Previous population-based studies quantified these effects using estimates of direct and indirect costs and productivity-adjusted life years lost [5, 19]. As diabetes prevalence is projected to rise [4], the economic burden is likely to increase. Given a mean age at diagnosis of approximately 53 years in Germany [21], our estimates of productivity losses associated with five additional years of diabetes duration using data from the early stage of the disease probably underestimate the average productivity loss in the whole population with diabetes. These findings highlight the necessity for dual-targeted interventions, with one aimed at diabetes prevention and improvement of disease management, and the other focused on structural strategies addressing socio-economic disparities. To our knowledge, no interventions have specifically targeted diabetes-related productivity losses, especially among people in low SEP. However, effective glycemic control may prevent or delay complications and thereby mitigate productivity losses [22]. Future investigations and efforts aimed at reducing the productivity burden of diabetes should therefore account for the substantial role of SEP in diabetes-related productivity losses.

## Strengths & limitations

A major strength of our study lies in its large sample size and longitudinal design, allowing multiple assessments of individuals over time. Participants were followed for up to six interview waves, with a mean of two follow-ups per participant, corresponding to an average observational period of four years.

The validity of our analyses relies on several assumptions. First, we assume that self-reported diabetes status is correct. Once reported as present, we treated it as fixed for all subsequent follow-ups, even when later data were contradictory. This may lead to misclassification because an incorrect report of diabetes would persist across all subsequent follow-ups. Another potential source of misclassification is the presence of people with undiagnosed diabetes in the group of people without diabetes. The direction of bias is uncertain as healthier individuals – who are more likely to participate in labour force and tend to exhibit lower levels of SLD – may consult doctors less frequently and thus be more likely to remain undiagnosed, or on the other hand may attend screenings more often, leading to earlier detection. Additionally, since diabetes status was assessed biennially, diabetes duration may be underestimated, potentially contributing to an overestimation of the true effects. Moreover, the observed negative effect of a five-year increase in diabetes duration on LFP and SLD may be influenced by our analyses being restricted to early post-diagnosis years. Effects may differ among individuals with longer disease history. Second, we assume that the included time-varying confounders sufficiently adjust for confounding and selection bias due to loss to follow-up. This assumption implies that we have complete and accurate data for all time-varying confounders. For self-reported SLD, which are used as outcome or previous outcome in the role of a time-varying confounder, recall bias may be present and reduce the accuracy of the variable. Although adjustment for additional confounders such as diet, stress and comorbidities would have been desirable, data on these variables were unavailable or too sparse. As we assume a high correlation between these omitted factors and the included confounders, we believe that the included variables at least partially adjusted for confounding from the omitted factors. The possibility of residual confounding arising from their exclusion cannot be ruled out. However, our sensitivity analyses suggest that estimates for LFP-shortfall are robust to a moderate degree of unmeasured confounding (see supplementary material). Third, we suppose correct specification of the models for diabetes onset and censoring, conditional on prior information. Despite substantial improvement after IPTC weighting, variable imbalance was still present for time-varying confounders, as shown in the supplementary material. Nevertheless, not accounting for potential time-dependent confounding resulted in effect estimates similar to those from the weighted analyses (supplementary material). Taken together, these findings suggest that any remaining bias from residual confounding is likely to be small. Finally, we assume that our MSMs estimating the effect of diabetes duration on the probability of LFP and the number of SLD, within levels of baseline variables, are also correctly specified.

A detailed discussion on the derivation of SEP, its use as a composite measure and potential biases arising from self-reported diabetes and the non-distinction between diabetes types is provided in our earlier work [18].

Observations were mainly excluded from our analyses due to missing information on key variables. Individuals with such missing information were treated as right-censored and subsequent follow-up data were excluded. Although, interval-censoring may be more appropriate than right-censoring in our study, we are unaware of established methods that handle interval-censoring while adjusting for time-varying confounding in repeated outcome data. Data exclusion caused substantial missing data and markedly declining participation rates across successive follow-ups. Additionally, the exclusion of prevalent cases at baseline further reduced the sample size, in particular among individuals with present diabetes and within strata of baseline variables at later follow-ups. While stabilized IPTC weights account for selection bias, these factors contributed to a loss of statistical power, affecting the precision of our estimates. Since the GSOEP is still ongoing, future studies may replicate our analyses with larger sample size, especially for the later follow-ups considered here. Notably, imputed-data sensitivity analyses supported the key findings of our main analysis and yielded more precise estimates. However, results across age and sex subgroups could not be fully replicated. Future research using alternative datasets could further evaluate both the confounders included in our models and additional factors not considered here.

## Conclusion

In summary, this study showed that diabetes-associated productivity losses are disproportionally higher among people in low SEP. These findings underscore the urgent need for targeted interventions in diabetes prevention, disease management and strategies reducing socio-economic disparities.

## Supplementary Material

ckag041_Supplementary_Data

## Data Availability

The SOEP-Core data can be ordered for scientific purposes via the research data center of the SOEP at DIW Berlin. Details on the procedure can be found at https://www.diw.de/en/diw_01.c.601584.en/data_access.html.
