# Supplementary material for: Causal effect of diabetes duration on productivity by socio-economic position in Germany between 2009 and 2021
Source: Eur J Public Health. 2026 Mar 24;36(2):ckag041. doi: 10.1093/eurpub/ckag041 (PMC13010310; doi:10.1093/eurpub/ckag041)
Supplement: ckag041_Supplementary_Data [file ckag041_supplementary_data.docx]

**Supplementary material**

**Description of diabetes duration**


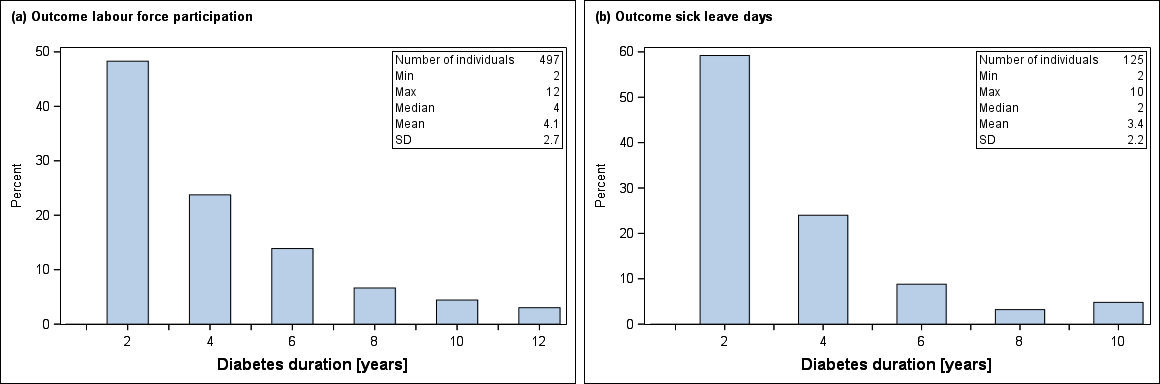


Figure S1. Distribution and descriptive statistics of diabetes duration at the last individual follow-up among participants with diabetes in study populations defined by the outcomes labour force participation (a) and sick leave days (b).
Alt text: Composite graphic with two histograms illustrating the distribution of diabetes duration in years. The left panel represents the study population for the outcome labour force participation, the right panel represents the study population for the outcome sick leave days. Each panel includes information on sample size, minimum, maximum, median, mean and standard deviation.

**Calculation of stabilized inverse-probability-of-treatment-and-censoring weights**

Stabilized inverse-probability-of-treatment-and-censoring (IPTC) weights were calculated by multiplying stabilized inverse-probability-of-treatment (IPT) and stabilized inverse-probability-of-censoring (IPC) weights, each obtained through pooled logistic regression. The numerator of the stabilized IPT weights represents the probability of the persons’ diabetes history at follow-up *t*, given their past diabetes history, baseline year and variables, and follow-up interview indicator *t*. The denominator was derived in a similar way, additionally including the time-varying confounders *CV(t)* as a conditioning factor.

$$IPT= \prod_{t=1}^{T} \frac{Pr\left( D(t)=d(t)|D(t-1)=d(t-1), CF=cf \right)}{Pr\left( D(t)=d(t)|D(t-1)=d(t-1), CV(t)=cv(t), CF=cf \right)}$$

To calculate stabilized IPC weights, the probability for remaining uncensored at *t+1* was modelled equivalently, though including diabetes status at *t* as a further confounder.

$$IPC=\prod_{t=1}^{T} \frac{Pr\left( C(t+1)=0|C(t)=0, D(t) = d(t), CF=cf \right)}{Pr\left( C(t+1)=0|C(t)=0, D(t) = d(t), CV(t)=cv(t), CF=cf \right)}$$

The inclusion of stabilized IPTC weights effectively controls for confounding by generating a pseudo-population in which *CV(t)* no longer acts as a confounder, while preserving the causal relationship between diabetes duration and labour force participation (LFP) or sick leave days (SLD) as observed in the original study population.

While SLD, body mass index (BMI) and socio-economic position (SEP) were included as linear terms, age was modelled as a cubic spline with three knots placed at the stabilized IPTC-weighted empirical 10^th^, 50^th^ and 90^th^ quantiles of the baseline age distribution. We used the SAS macro RCSPLINE developed by Frank Harrell for spline estimation. IPT and IPC weights were obtained using the PROC LOGISTIC procedure in SAS.

**Sensitivity analyses**

***Methods***

To assess the stability and distribution of the stabilized IPTC weights, we calculated the mean, standard deviation (SD) and range, following the approach suggested by Cole and Hernán [1]. This analysis aimed to determine if the mean values deviated from unity or exhibited extreme values.

The performance of the stabilized IPTC weights was evaluated by computing absolute standardized differences (ASDs) for the time-varying confounders BMI, previous outcome, physical activity and smoking according to the method described by Yang and Dalton [2]. These ASDs were used to assess variable balance across different levels of diabetes. The SAS macros STDDIFF and LOVE were employed for standardized difference calculations [2] and graphical illustration of variable balance [3], respectively.

Results from the marginal structural models (MSMs) were compared to those from crude regression models to evaluate the impact of adjusting for time-varying versus time-fixed confounding and the influence of BMI, physical activity, smoking and previous outcome on effect estimates. The first crude model included the time-fixed baseline variables *CF*, diabetes duration, follow-up interview indicator *t* and baseline year as independent variables. In the second model baseline values of the time-varying confounders *CV(t), i.e. CV(t=0),* were added to the baseline variables *CF*. Robust variance estimation was used to account for within-subject correlation.

To evaluate the robustness of our findings to unmeasured confounding, we employed the E-value method introduced by VanderWeele and Ding [4].

The LFP study population included data collected between 2009 and 2021. To assess the potential impact of the COVID-19 pandemic, we repeated the analysis after excluding data from 2020 and 2021. Because SLD were reported retrospectively, the SLD study population contained only observations from 2009 to 2019. Therefore, it was not possible to perform a sensitivity analysis related to the COVID-19 pandemic for this study population.

We performed multiple imputation using the fully conditional specification method to investigate the impact of declining participation over increasing follow-up waves. For categorical variables, the discriminant method was used. For continuous variables, the predictive mean method was applied. The dataset for imputation contained individuals without diabetes at baseline, aged 20–69 years and residing in private households. The imputation model included the variables with missing data, which were BMI, physical activity, smoking status, diabetes and LFP or SLD, in addition to the baseline variables sex, age, migration background and SEP. Imputations were carried out using the SAS procedures PROC MI and PROC MIANALYZE, following the approach described by Berglund [5]. Since stabilized IPT weights reached values as high as 706.95, but the 99^th^ percentiles of the empirical weight distributions were below 1.75, we applied weight trimming at 1.75 to improve estimate precision. Similarly, because the 1^st^ percentiles of the empirical weight distributions were above 0.5, but minimum values were as small as 0.02, we set stabilized IPT weights below 0.5 to the value of 0.5 [1].

***Results***

The overall mean (SD) of the stabilized IPTC weights was equal to 1.00 (0.22) in the LFP study population with a range of 0.04 to 8.85. Accordingly, the overall mean (SD) of the stabilized IPTC weights was equal to 1.00 (0.19) in the SLD study population with a range of 0.05 to 6.32. The mean weight values for individual follow-ups varied between 0.99 and 1.20, with corresponding SDs ranging from 0.09 to 0.77. The stability of the weights appears to decline over time, as both deviations from unity and SDs progressively increase. The absence of extreme values, combined with mean estimates remaining close to unity and relatively small SDs, suggests no substantial evidence of model misspecification.

The analysis of ASDs showed a substantial improvement in variable balance through weighting, with one exception (see Figure S2). For the continuous variable BMI included in *CV(t)*, the maximum ASD decreased from 0.92 to 0.38 after weighting. The ASD for previous SLD remained stable at approximately 0.17. For the categorical variables physical activity and smoking included in *CV(t)*, ASDs were reduced by roughly 50% on average, with the maximum post-weighting ASD being 0.30. However, in the case of previous LFP, weighting led to a slight increase in ASD from 0.37 to 0.40.


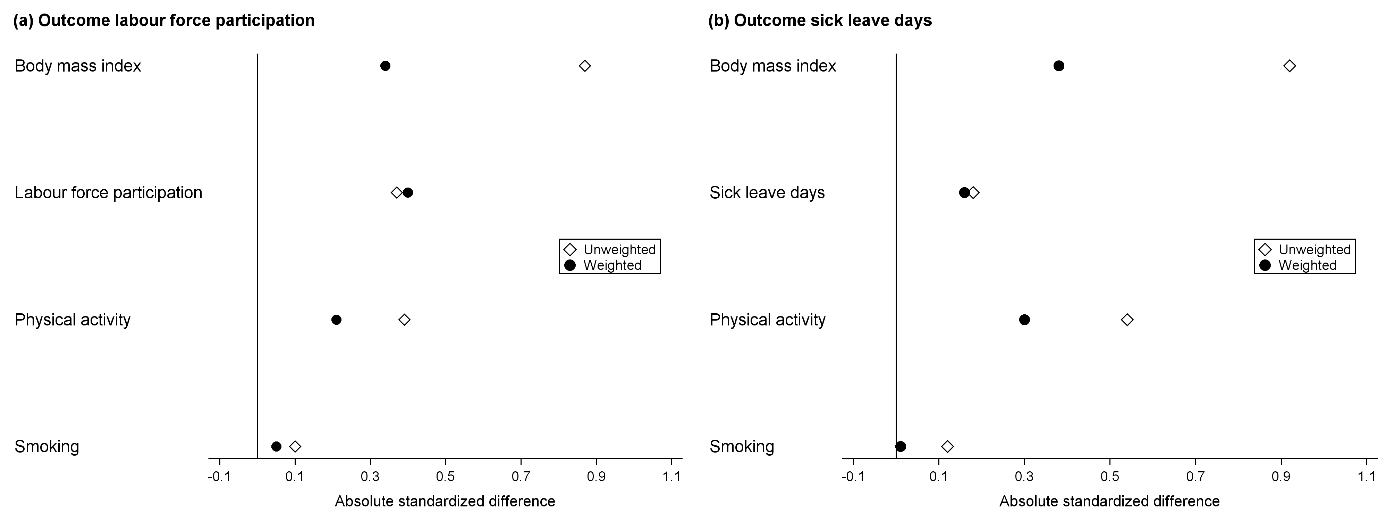


Figure S2. Absolute standardized differences for the time-varying confounders body mass index, previous outcome, physical activity and smoking in study populations defined by the outcomes labour force participation (a) and sick leave days (b).
Alt text: Composite graphic with two love plots comparing absolute standardized differences before and after weighting for body mass index, previous outcome, physical activity and smoking. The left plot represents the study population for the outcome labour force participation, the right plot the study population for the outcome sick leave days.

The crude Poisson regression, which included baseline variables *CF*, diabetes duration, follow-up interview indicator *t* and baseline year as independent variables, estimated a LFP-shortfall of 13.5% (95% confidence interval, CI: 7.2;19.3). In the second model, where *CV(t=0)* was added to *CF*, the estimated LFP-shortfall was 10.4% (95% CI: 5.2;15.2). In comparison, when time-varying confounding was addressed using marginal structural Poisson regression, the estimated shortfall was 13.8% (95% CI: 5.8;21.1). The absolute increase in SLD was estimated to be 8.5 (95% CI: -1.9;18.9) using crude linear regression including baseline variables *CF*, diabetes duration, follow-up interview indicator *t* and baseline year as independent variables. When *CV(t=0)* was additionally included, the estimate was 6.8 SLD (95% CI: -3.9;17.5). Marginal structural linear regression produced an estimate of 6.8 (95% CI: -5.4;19.0). These findings indicate that the inclusion of the time-varying confounders has a minor impact on effect estimates. The confidence intervals from the MSMs were wider than those from the crude models, suggesting that adjustment for time-varying confounding introduced additional variability, resulting in wider and thus more conservative confidence intervals.

Using the E-value formula, we calculated a value of 1.6 for the estimated relative risk (RR) of 0.86 (95% CI: 0.79;0.94) within the LFP study population. This implies that an unmeasured confounder would need to be associated with both diabetes and LFP by an average RR of 1.6, conditional on the selected variables, to fully explain the observed RR. An average RR of 1.3, resulting from the upper confidence limit, could shift the CI to include the null. Concluding, the estimate appears moderately robust, suggesting moderate evidence for a causal relationship. For the SLD study population, the E-value was 2.2 for the estimated RR of 1.41 (95% CI: 0.86;2.31), and 1.0 for the lower confidence limit. This indicates that no unmeasured confounding would be necessary to move the CI to include the null, suggesting weak evidence for a causal effect. These findings are consistent with the results presented in Figure 2b, where the overall CI included the null value.

The exclusion of data from 2020 and 2021 had a minor impact on the LFP-shortfall estimates, indicating that the COVID-19 pandemic had negligible impact on our results. The overall relative shortfall was 12.4% (95% CI: 2.7;21.0) compared with 13.8% (95% CI: 5.8;21.1) in the main analysis. The SEP-related gradient was larger than in the main analysis, with estimates of 20.6%, 12.6% and 1.4% for low, middle and high SEP, respectively, compared with 18.3%, 13.9% and 8.1% in the main analysis. All 95% CIs were wider than those in the main analysis due to the smaller sample size. Details can be found in Figure S3.


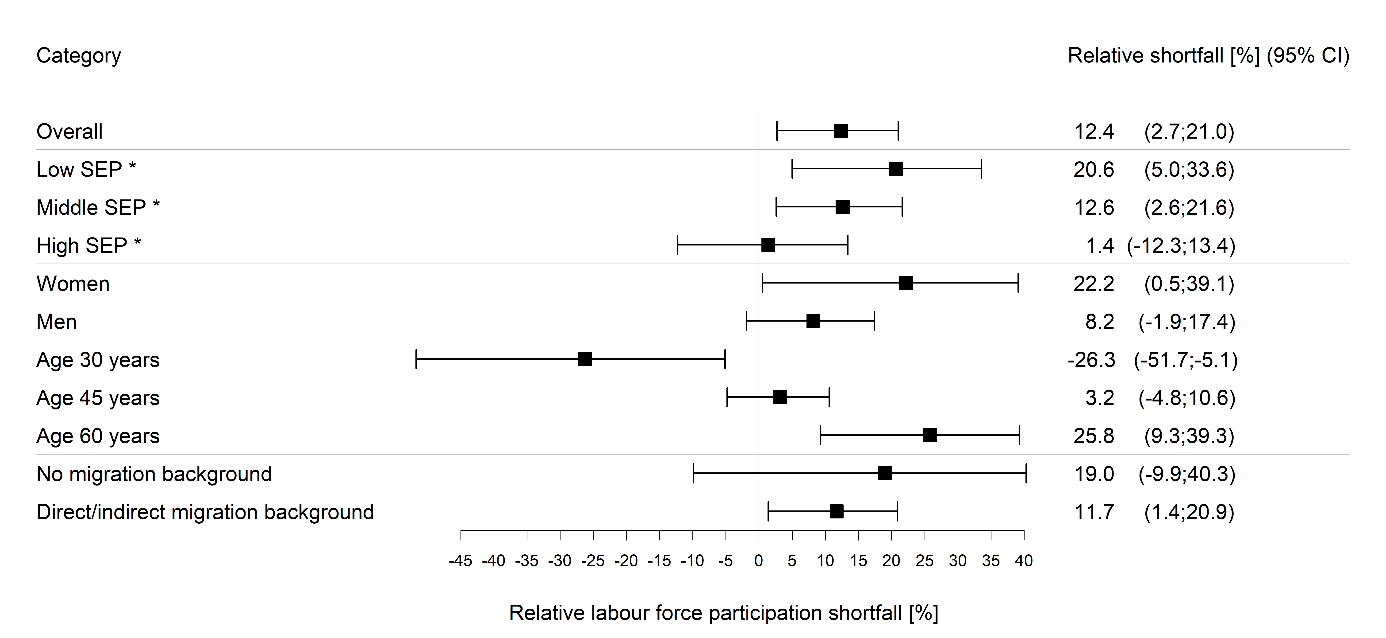


Figure S3. Relative labour force participation shortfall associated with five additional years of diabetes duration overall and by subgroups. Results are based on marginal structural regression models for repeated measures adjusted for time-fixed and time-varying confounding using Poisson regression on data excluding observations from 2020 and 2021.

* SEP, socio-economic position.

Alt text: Forest plot showing the relative labour force participation shortfall in percent with 95 percent confidence intervals overall and for subgroups defined by socio-economic position, sex, age and migration background.

Multiple imputation resulted in an analysis sample of 30 646 individuals with six follow-ups each for the LFP study population. As shown in Figure S4a, the overall effect estimate of 11.1% (95% CI: 7.0;15.3) was slightly lower than in the main analysis (13.8% (95% CI: 5.8;21.1)), but showed a considerably narrower CI. Although estimates for SEP subgroups were smaller, the differences between SEP groups were more pronounced, reinforcing the gradient observed in the main analysis. Women exhibited a higher estimate of LFP-shortfall compared to men, but the difference between sexes was less pronounced than in the main analysis, which indicated more than double the shortfall among women than among men. The age-related gradient was weaker in the imputed sample, with all effect estimates remaining above zero. While estimates stratified by migration background were somewhat lower than in the main analysis, no substantial differences in LFP-shortfall between the two strata were observed, aligning with the main results.

After multiple imputation, the analysis sample for the SLD study population comprised 20 814 individuals, each with five follow-ups. Results derived from the imputed dataset showed a slightly lower effect estimate of 5.8 (95% CI: 1.9;9.8) compared to the main findings (6.8 (95% CI: -5.4;19.0)), while confidence intervals were notably narrower (Figure S4b). Although a decreasing trend in SLD could be observed with increasing SEP, the range of estimates between SEP groups was smaller, with values of 7.4 (95% CI: 1.0;13.8) for the highest and 4.4 (95% CI: 1.9;9.8) for the lowest SEP group. Estimates were similar across sex and age groups, in contrast to the trends in the main analysis. The lack of a clear difference between migration background was concordant with the main findings.


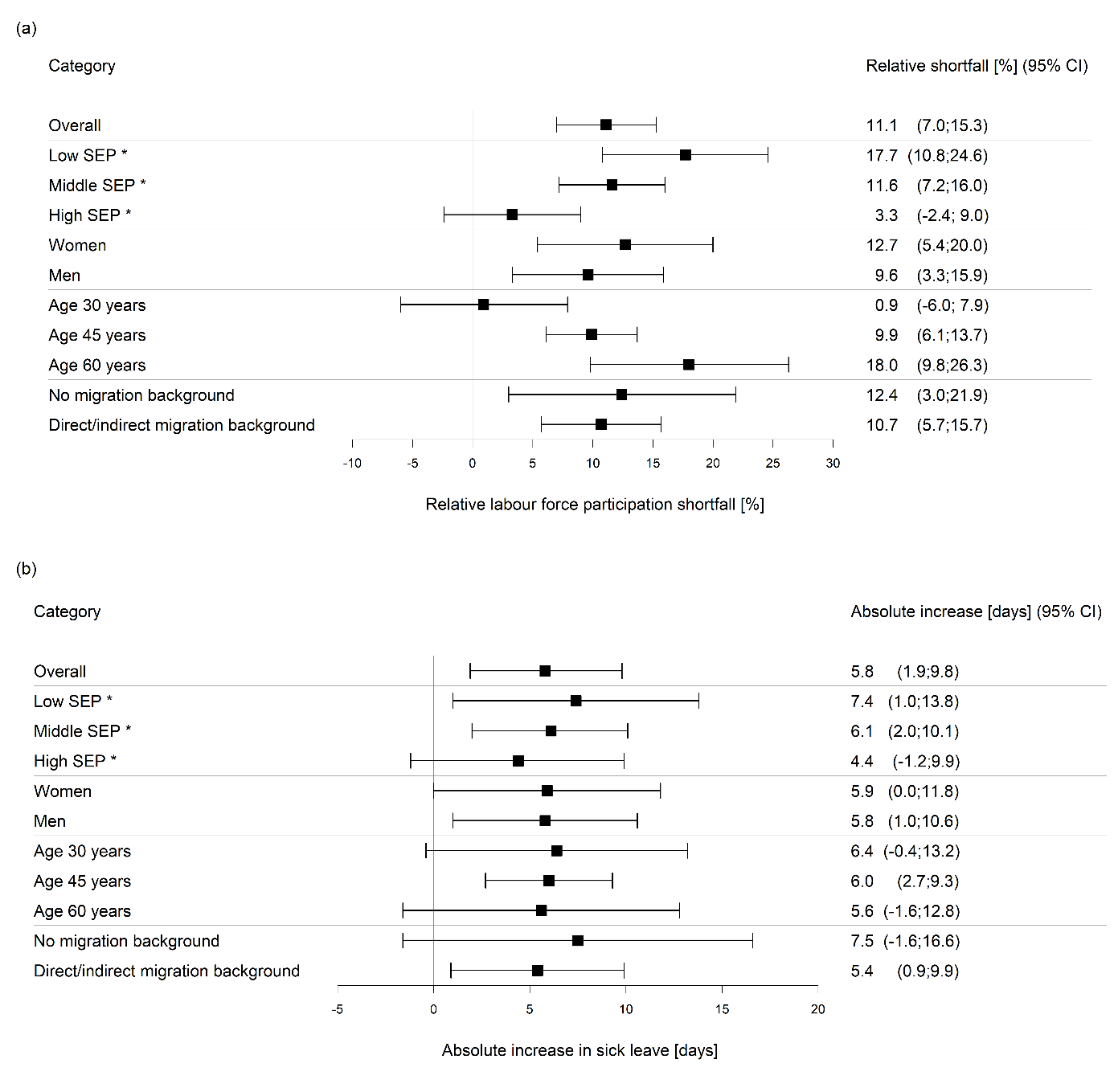


Figure S4. Relative labour force participation shortfall (a) and absolute increase in sick leave days (b) associated with five additional years of diabetes duration overall and by subgroups. Results are based on marginal structural regression models for repeated measures adjusted for time-fixed and time-varying confounding using Poisson regression (a) and linear regression (b) on the imputed datasets.

* SEP, socio-economic position.

Alt text: Composite graphic with two forest plots. The upper plot shows the relative labour force participation shortfall in percent with 95 percent confidence intervals. The lower plot shows the absolute increase in sick leave days with 95 percent confidence intervals. Both plots present results overall and for subgroups defined by socio-economic position, sex, age and migration background.

**References**

1 Cole SR, Hernán MA. Constructing inverse probability weights for marginal structural models. *Am J Epidemiol* 2008;**168**:656–64.

2 Yang D, Dalton JE. A unified approach to measuring the effect size between two groups using SAS®. *Proceedings of the SAS Global Forum 2012 Conference. Orlando, Florida: SAS Institute Inc.* 2012.

3 GitHub. Easy SAS love plot for covariate imbalance. https://github.com/alankinlaw/Easy-SAS-Love-Plot-for-Covariate-Imbalance (30 January 2026, date last accessed).

4 VanderWeele TJ, Ding P. Sensitivity Analysis in Observational Research: Introducing the E-Value. *Ann Intern Med* 2017;**167**:268–74.

5 Pan W. Akaike’s Information Criterion in Generalized Estimating Equations. *Biometrics* 2001;**57**:120–25.
